# Small Extracellular Vesicles as Biomarkers in Sarcoma Follow-Up: Protocol for a Prospective, Multicentric Pilot Study

**DOI:** 10.2196/63718

**Published:** 2025-09-09

**Authors:** Valentin Vautrot, Alice Hervieu, Aurélie Bertaut, Céline Charon-Barra, Isen Naiken, Sylvain Causseret, Loic Chaigneau, Isabelle Desmoulins, Emilie Rederstoff, Nicolas Isambert, Jessica Gobbo

**Affiliations:** 1 Department of Medical Oncology Early Phase Unit Georges-François Leclerc Centre Dijon France; 2 Unit of Methodology Biostatistics and Data Management Georges-François Leclerc Centre Dijon France; 3 Unit of Pathology Department of Biology and Pathology of Tumours Georges-François Leclerc Centre Dijon France; 4 Georges-François Leclerc Centre Dijon France; 5 University Hospital of Besançon Jean Minjoz Besançon France; 6 Department of Medical Oncology Georges-François Leclerc Centre Dijon France; 7 Department of Oncology Pôle Régional de Cancérologie University Hospital of Poitiers Poitiers France; 8 INSERM, Centre d'Investigation Clinique 1402 Poitiers France; 9 PRODICET UR24144 Poitiers France; 10 HSP-pathies Team, Label Ligue National contre le Cancer INSERM UMR 1231 Dijon France; 11 Faculty of Medicine and Pharmacy Université Bourgorgne Europe Dijon France; 12 Module Plurithématique INSERM, CIC1432 Dijon France

**Keywords:** pilot study, liquid biopsy, small extracellular vesicle, sarcoma, cancer monitoring

## Abstract

**Background:**

Sarcomas are rare cancer with a heterogeneous group of tumors. They affect both genders across all age groups and present significant heterogeneity, with more than 70 histological subtypes. Despite tailored treatments, the high metastatic potential of sarcomas remains a major factor in poor patient survival, as metastasis is often the leading cause of death. Currently, metastatic risk assessment relies mainly on histological grading; yet, this method has limitations due to the disease’s heterogeneity. Advances in genomic and transcriptomic research have identified potential molecular signatures, but these approaches lack reproducibility and prognostic reliability. Therefore, new biomarkers are essential for improving risk prediction and therapy adaptation. Recent studies highlight that sarcoma cells secrete extracellular vesicles, particularly small extracellular vesicles (sEVs). These nanovesicles, abundant in bodily fluids such as blood, urine, and saliva, play a crucial role in tumor development, growth, and metastasis. sEVs contain proteins and nucleic acids that mirror tumor characteristics. Given their presence in blood, sEVs offer a promising avenue for noninvasive molecular cancer analysis via liquid biopsy. Preliminary studies in Ewing sarcoma have shown substantial alterations in sEV-derived transcripts, underscoring their potential in tracking disease progression and treatment efficacy.

**Objective:**

This study aims to investigate whether sEVs can serve as reliable biomarkers for monitoring sarcoma progression and predicting recurrence risk.

**Methods:**

This prospective, multicentric pilot study will enroll adult patients diagnosed with localized or metastatic liposarcomas, leiomyosarcomas, or undifferentiated pleomorphic sarcomas at 3 French cancer centers. The study’s primary goal is to quantify sEVs and analyze their protein and RNA content in the blood of patients with localized or metastatic sarcomas before and after the treatment. sEVs will be isolated from plasma samples, and protein and microRNA concentration will be determined. Research will last, on average, 6 months for patients with localized sarcoma and 4 months for patients with metastatic sarcoma.

**Results:**

We expect to identify differences in exosome levels based on disease stage and observe correlations between exosome dynamics and treatment response. If confirmed, these findings could establish sEVs as noninvasive biomarkers for monitoring therapy effectiveness and disease progression in patients with sarcoma.

**Conclusions:**

This study could establish a novel, noninvasive biomarker for sarcoma prognosis and treatment monitoring. If successful, a nationwide study will be launched to confirm findings in a larger patient cohort, potentially revolutionizing sarcoma management and improving patient outcomes.

**Trial Registration:**

ClinicalTrials.gov NCT03800121; https://clinicaltrials.gov/ct2/show/study/NCT03800121

**International Registered Report Identifier (IRRID):**

DERR1-10.2196/63718

## Introduction

Sarcomas are rare cancers representing 1%-2% of all adult cancers with more than 5000 cases per year in France recently [1]. They affect men and women of all ages and constitute a heterogeneous group of tumors, with more than 70 different histological types and subtypes [2]. Despite adapted therapies, the metastatic risk of these diseases currently has a major negative impact on patient survival. Metastatic dissemination of tumor cells is responsible in most cases for patient death. The estimation of sarcoma’s metastatic risk is complex, given the histological heterogeneity of this pathology. It is therefore essential that, at diagnosis, a reliable evaluation of metastatic potential may be determined to better adapt the therapeutic strategy. Currently, the estimation of this risk is based essentially on histological criteria, namely the tumor grade. In recent years, large-scale genome and transcriptome analyses have allowed the identification of tumor molecular signatures predictive of prognosis. However, results reproducibility can be discussed, and the biological and prognostic significance remains limited. It is therefore necessary to find new biomarkers.

It has recently been found that sarcoma tumor cells secrete extracellular vesicles (EVs) [3] including small EVs (sEVs). In this study protocol, we will use the term exosomes to refer to small EVs. Exosomes are nanovesicles secreted by all the cells of the human body and found in abundance in different biological fluids such as blood, urine, saliva, and breast milk. Recently, the role and involvement of exosomes in sarcoma [4,5] development have been demonstrated [6]. Indeed, exosomes seem to play an important role in tumorigenesis, growth, tumor progression, and metastases appearance. Exosomes contain many proteins and nucleic acids (DNA, RNA, and microRNA), reflecting tumor characteristics. It has been shown that the amount of exosomes can be correlated with the grade of tumor malignancy [7,8]. Present in the blood, they offer the possibility of a noninvasive analysis of cancer cell molecular information by liquid biopsy. Recent preclinical studies have demonstrated strong changes in more than 1200 transcripts present in exosomes derived from Ewing sarcoma cells. Therefore, the study of plasma-derived exosomes in patients with sarcoma has a high potential to evaluate cancer pathogenesis, progression, and treatment efficacy.

The aim of this study is to demonstrate the potential of exosomes as biomarkers for monitoring disease progression and predicting treatment response in patients with both metastatic and nonmetastatic sarcoma.

## Methods

### Study Population

Adult patients with a diagnosed sarcoma, such as liposarcomas, leiomyosarcomas, or undifferentiated pleomorphic sarcoma (UPS) and managed at Georges-François Leclerc Center in Dijon or at Jean Minjoz Center in Besançon or CHU Poitiers.

### Study Objectives and Endpoints

The main objective of this pilot study is to quantify exosomes from plasma and to analyze protein and RNA content in patients with sarcoma, with localized or metastatic disease. Analysis will be performed before and after surgery in localized disease and before and after first-line chemotherapy in metastatic sarcoma. Some patients may have received neoadjuvant chemotherapy with or without radiotherapy.

The secondary objectives are to:

1. Determine whether the initial exosome concentration, protein, and RNA profile vary in the plasma with localized or metastatic stage of the disease.
2. Determine whether the exosome concentration as well as protein and RNA profile varies upon treatment.
3. Determine whether the initial exosome concentration (T0) is associated with a response to treatment.
4. Determine whether changes in exosome concentration before and after treatment are associated with a response to treatment.

The primary endpoint of the study is the quantification of exosome concentration in the blood, as well as the measurement of protein and RNA levels after exosome lysis. Measures will be performed before and after surgery in case of localized disease and before and after chemotherapy in case of metastatic disease.

The secondary endpoints will be exosome blood concentration, as well as protein and RNA concentrations measured after exosome lysis. Treatment response and tumor progression (locoregional or appearance of metastases) will be assessed by magnetic resonance imaging (MRI) or computed tomography (CT) scan using RECIST (Response Evaluation Criteria in Solid Tumors) V1.1 criteria.

### Study Design

**Figure 1:**
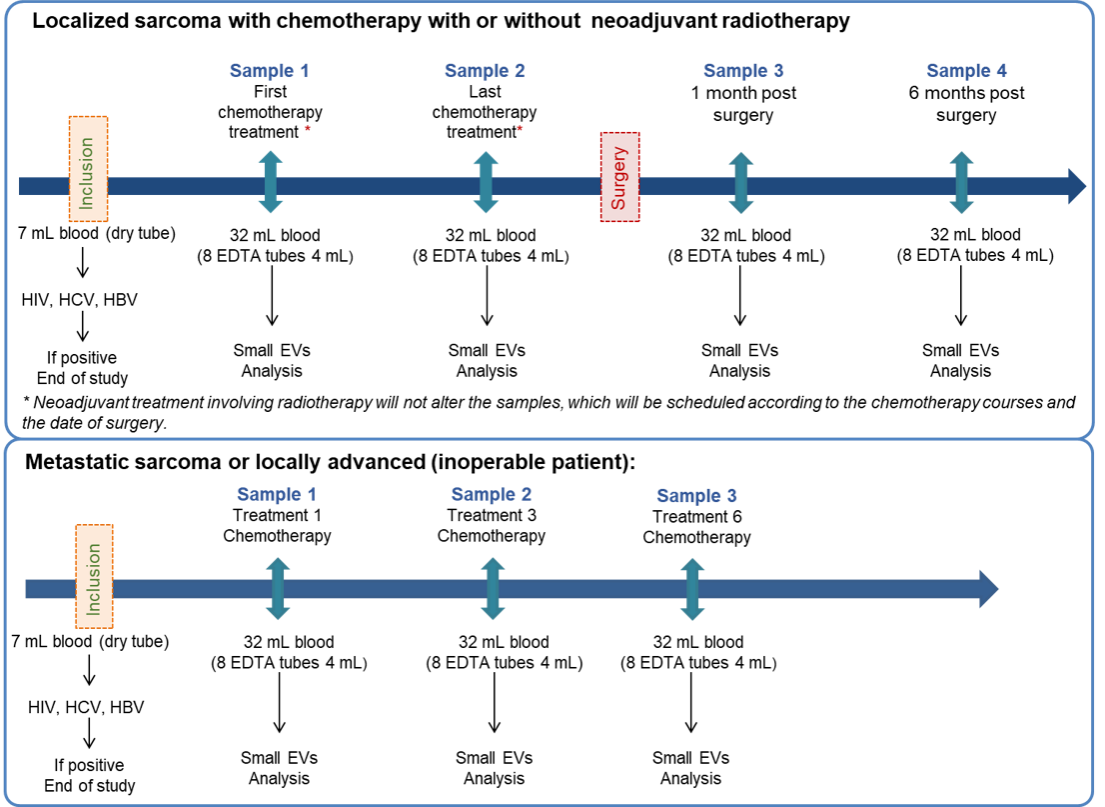
Study design. EDTA: ethylenediaminetetraacetic acid; EV: extracellular vesicle; HBV: hepatitis B virus; HCV: hepatitis C virus.

Pilot, prospective, multicentric, nonrandomized study, without direct individual benefit. The study is supported by the Institut National du Cancer (INCa) platform CLIP² (Certified Early-Phase Clinical Trial Centers) at the Georges-François Leclerc Center in Dijon, CHU Poitiers, and Jean Minjoz Center in Besançon. The study inclusion period will be 48 months and the total study duration will be 60 months. Study design is depicted in Figure 1. Research will last an average of 6 months for patients with localized sarcoma and 4 months for patients with metastatic sarcoma.

Investigators receive an informative notice on the EXOSARC (Study of Exosomes in Monitoring Patients With Sarcoma) study and will be provided samples of eligible patients. Upon written patient consent, serology to HIV, hepatitis C virus, and hepatitis B virus will be tested (Figure 1). Only patients with negative serology for these infectious agents will be included, to protect technicians from biological risks, in agreement with laboratory procedures. Exact inclusion and exclusion criteria are available in Textbox 1.

**EXOSARC (Study of Exosomes in Monitoring Patients With Sarcoma) inclusion and exclusion criteria.**

**Inclusion criteria**

- Men and women diagnosed with localized or metastatic soft tissue sarcoma (liposarcoma, leiomyosarcomas, or undifferentiated pleomorphic sarcomas [UPS]).
- Adult patients (≥18 years old) with histologically confirmed sarcoma, including but not limited to liposarcomas, leiomyosarcomas, and UPS.
- Previous treatment of the disease with chemotherapy, radiotherapy, or surgery is allowed if it has been completed for more than 12 months at the time of inclusion.
- Age ≥18 years old.
- For metastatic or locally advanced (inoperable) sarcoma, patients for whom first-line metastatic chemotherapy is indicated.
- Affiliated to French social security or beneficiary of such a regimen.
- Written informed consent.

**Exclusion criteria**

- Patient with other synchronous tumors
- Patient with sarcoma in irradiated tissue.
- Patient with a history of cancer other than sarcoma in the 5 years preceding sarcoma diagnosis.
- Patient unable to undergo medical follow-up for geographical, social or psychological reasons,
- Person benefiting from an adult protection system (including guardianship and trusteeship).
- Positive serology to HIV or hepatitis B virus or hepatitis C virus.
- Pregnant or lactating woman.
- Patients unable to understand, read, or sign an informed consent.

### Patient Identification and Recruitment

Patients will be identified and recruited from participating oncology centers through:

- Diagnosis codes (*ICD-10* [*International Statistical Classification of Diseases and Related Health Problems 10th Revision*]) and pathology reports from hospital databases.
- Referral by treating oncologists and multidisciplinary sarcoma teams based on eligibility criteria.
- Screening during routine clinical visits for patients with newly diagnosed sarcoma or those scheduled for treatment.

### Sampling

A total of 32 mL of blood (8× 4 mL EDTA [ethylenediaminetetraacetic acid] tubes) will be collected at each sampling for exosome analysis in both cohorts.

- For cohort 1 (patients with localized sarcoma and undoing neoadjuvant chemotherapy), blood samples will be collected before surgery at cure 1 (sample 1) and at the last cure preceding surgery (sample 2). Then, at 1 month (sample 3) and 6-8 months post surgery (sample 4).
- For cohort 2 (patients with metastatic or locally advanced inoperable sarcoma), a blood sample will be collected before initial chemotherapy administration (sample 1). Then, at the third (sample 2) and at the sixth chemotherapy treatment (sample 3).
- Note that sample collection will take place before chemotherapy treatment even if the cure schedule is changed or postponed. Samples will be kept for a maximum of 2 hours at 4 °C.

### Blood Collection and Storage

Plasma will be collected by centrifugation of blood samples (EDTA tube; 2200 rpm for 15 minutes at 4 °C). All the 3 components obtained will be aliquoted and stored on site at −80 °C.

### Extracellular Vesicle Analysis

Thawed plasma samples will be processed to centrifugation at 2000 g at 4 °C for 20 minutes. Exosomes will be extracted with Total sEV Precipitation Reagent (4484450, Thermo Fisher Scientific) for 10 minutes following homogenization. Part of the EVs will be used to determine their concentration and distribution profile using Nanotracking Analysis (NS300, Malvern). The data will be analyzed using Nanosight NTA 3.2 Analytical Software (Malvern Instruments). The other part will be used for the analysis of EV contents (proteins and RNA) and stored at –80 °C. Content analysis (proteins and RNA) will be performed using Western blot analysis, enzyme-linked immunosorbent assay, and RNA sequencing (Illumina) approaches. It is expected that all collected blood will be used for extracellular vesicles and content analysis.

### Data Collection

The following data will be collected during the study, at the time of inclusion, and during medical follow-up: demographic and clinical data (age, sex, weight, height, performance status, etc), medical history, date of diagnosis, first-line treatment, disease evaluation reports (biological, imaging, etc), exosome analysis data (total exosome concentration, HSP70 exosome concentration, and multiple miRNA dosing). These data will be sent as a standard report from the laboratory in charge of these analyses (Inserm UMR1231). All data will be recorded in a Case Report Form (CRF).

### Sample Size

A total of 30 patients, for whom all samples required for the study have been collected, should be included, distributed as follows:

1. Group 1: 15 patients with localized disease, for whom the 4 samples required for the study were collected.
2. Group 2: 15 patients with metastatic disease, for whom the 3 samples required for the study were collected.

These numbers will provide preliminary data before considering a larger study. The primary objective of this study is to measure the markers of interest in patients. Given that the study focuses on rare cancers and that statistical analyses are only secondary objectives, no statistical power calculation has been performed for this study. Patients for whom the total number of required samples was not achieved will be replaced.

### Statistical Analysis

Plasma exosomes as well as exosomal protein and RNA concentrations will be described by their mean (SD) and their median (with extent). Comparison of baseline concentrations of participants with localized disease versus metastatic disease will be performed using nonparametric Wilcoxon tests. Comparison of pretreatment and posttreatment (surgical or drug) concentrations will be performed using nonparametric tests for matched data. Analyses will be carried out by subgroup (metastatic and localized) and on the whole population. Relative exosome variation before and after treatment will be compared in responders and nonresponders using nonparametric Wilcoxon tests. A Bonferroni correction will be applied to the *P* value to adjust the significance threshold during multiple comparisons.

The area under the receiver operating characteristic curve (AUC) modeling the response to treatment in function of initial exosome concentration will be plotted. AUCs with 95% CI values will be provided. The model will be adjusted to the type of disease (localized or metastatic). An exosome concentration threshold, predictive of response to treatment, will be determined. Performance (ie, sensitivity, specificity, and predictive values) associated with this threshold will be calculated. A similar approach will be followed concerning exosome concentration after treatment and delta between initial and posttreatment values. Different protein and RNA-derived markers will be analyzed. Performance associated to the absence or presence of these markers to predict response to treatment will be determined for each of the markers. Analyses will be performed under the software SAS 9.4 (SAS Institute).

### Patient and Public Involvement

The patient committee of the National League Against Cancer (“Comité de patients en recherche clinique en cancérologie de la Ligue contre le cancer”) was consulted on the information notice that will be given to patients asked to participate in this trial. This committee brings together patients, former patients, or patient relatives who have a shared interest in oncology medical research. The purpose of this consultation was to read and improve the patient information notice on the design and aim of the study, so that patients can clearly understand and decide whether to participate, in agreement with the principle of informed consent.

### Ethical Considerations

This study was approved by the local ethics committee and was authorized by the French Health Agency (ANSM [Agence Nationale de la Sécurité du Médicaments et des produits de santé], number 2018-A01393-52) in May 2018, and by the French Ethical Research Committee (CPP [Comité de Protection des Personnes], Ile de France 1) in October 2018. The clinical trial has been registered at ClinicalTrials.gov with the identifier NCT03800121. Last version of the study protocol dated May 19, 2021, is 1.4. Participants must provide informed consent in agreement with the Declaration of Helsinki. Participants will be informed of the objectives of the project and the risks and benefits of the explorations to be carried out. None of the tests will pose risks that could endanger participants’ lives. Confidentiality of participant data will be guaranteed at all times in agreement with the **National Commission on Informatics and Liberty** MR01 reference methodology registered for CGFL (1878714v0, July 30, 2015).

## Results

This study is expected to provide valuable insights into the role of exosomes in sarcomas, both in localized and metastatic stages. We anticipate that exosome concentration, as well as protein and RNA profiles, will differ between localized and metastatic disease, reflecting distinct tumor biology. Furthermore, we expect to observe variations in exosome concentration and content before and after treatment, potentially correlating with treatment response. A higher baseline exosome concentration may be associated with a poorer or more aggressive disease profile, while changes in exosome levels following treatment could serve as predictive biomarkers for therapeutic efficacy. The integration of exosome analysis with imaging-based assessments (MRI or CT scan using RECIST V1.1 criteria) will help establish correlations between exosome dynamics and tumor progression. If confirmed, these findings could pave the way for the use of exosomes as noninvasive biomarkers for monitoring sarcoma treatment response and disease progression, ultimately improving patient management.

## Discussion

### Anticipated Findings

As of 2025, despite the strong diagnostic and prognostic potential of exosomes in sarcomas [3,7-10], no prospective study has yet demonstrated the feasibility and clinical relevance of this concept. Our team has recently patented a technique to detect exosomes in various biological fluids, such as blood and urine [5] (patent WO/2015189395A1, Inserm Transfer). We have also launched a study on exosomes from solid tumors, analyzing their protein content and microRNA (Exodiag, NCT02662621), which has confirmed the technical feasibility of exosome research in humans. Notably, this study is currently the only ongoing clinical trial on sarcomas listed on ClinicalTrials.gov. Consistent with preclinical findings, we believe that studying plasma exosomes in rare cancers like sarcomas could significantly improve therapeutic management.

Looking ahead to 2030, our innovative approach has the potential to offer a noninvasive method for predicting treatment response and disease progression. If our pilot study yields positive results, we plan to conduct a nationwide study to validate these findings in a larger cohort.

### Conclusion

This pilot study proposes an innovative approach to monitoring patients with sarcoma by evaluating the potential of sEVs as noninvasive biomarkers. By analyzing the concentration and molecular content (proteins and RNA) of circulating exosomes, the study aims to improve our understanding of sarcoma biology in both localized and metastatic settings and to correlate these markers with treatment response. The expected outcomes could enhance prognostic assessment and therapeutic monitoring, ultimately enabling more personalized patient management. If successful, this pilot study will lay the groundwork for a larger nationwide clinical trial, with the potential to significantly advance the clinical care of patients with sarcoma.

## Abbreviations

ANSM: Agence Nationale de la Sécurité du Médicaments et des produits de santé
AUC: area under the receiver operating characteristic curve
CLIP²: Certified Early-Phase Clinical Trial Centers
CPP: Comité de Protection des Personnes
CRF: Case Report Form
CT: computed tomography
EDTA: ethylenediaminetetraacetic acid
EV: extracellular vesicle
EXOSARC: Study of Exosomes in Monitoring Patients with Sarcoma
ICD-10: International Statistical Classification of Diseases and Related Health Problems 10th Revision
INCa: Institut National du Cancer
MRI: magnetic resonance imaging
RECIST: Response Evaluation Criteria in Solid Tumors
sEV: small extracellular vesicles
UPS: undifferentiated pleomorphic sarcoma

## Data Availability

The investigator undertakes, on behalf of himself and all individuals involved in the conduct of the trial, to ensure the confidentiality of all information provided by the Centre Georges – François Leclerc until the publication of the trial results. This confidentiality obligation shall not apply to information that the investigator is required to disclose to patients as part of their participation in the trial, nor to information that has already been published. The investigator commits not to publish, disclose, or use, in any manner whatsoever, directly or indirectly, any scientific or technical information related to the trial. Any partial or total reproduction of this document is prohibited without the authorization of the Centre Georges François Leclerc. However, in accordance with Article R 5121-13 of the French Public Health Code, the center and the investigator may provide information related to the trial to: the Minister of Health, public health inspector physicians, public health inspector pharmacists, the Director General and inspectors of the ANSM (French National Agency for Medicines and Health Products Safety). The trial shall not be subject to any written or oral comments without the sponsor’s approval (CGFL); all information communicated or obtained during the trial shall be the exclusive property of the sponsor, who shall have full discretion over its use. At the end of the study, research-related documents (Investigator File, completed and validated CRFs, and other research documents) will be archived by the sponsor and the investigator sites in accordance with Article 2 of the decree of August 11, 2008, which sets the retention period at 15 years.
